# Scavenger Receptor Class B type 1 (SR-B1) and the modifiable risk factors of stroke

**DOI:** 10.1186/s41016-019-0178-3

**Published:** 2019-12-17

**Authors:** Cameron Lenahan, Lei Huang, Zachary D. Travis, John H. Zhang

**Affiliations:** 1 Burrell College of Osteopathic Medicine, Las Cruces, NM 88003 USA; 2 Center for Neuroscience Research, School of Medicine, Loma Linda University, Loma Linda, CA 92324 USA; 3 Department of Neurosurgery, School of Medicine, Loma Linda University, Loma Linda, CA 92350 USA; 4 Department of Physiology & Pharmacology, School of Medicine, Loma Linda University, Loma Linda, CA 92350 USA; 5 Department of Earth and Biological Sciences, School of Medicine, Loma Linda University, Loma Linda, CA 92350 USA; 6 Department of Anesthesiology, School of Medicine, Loma Linda University, Loma Linda, CA 92324 USA

**Keywords:** Scavenger Receptor Class B type 1, SR-B1, Atherosclerosis, Coronary heart disease, Diabetes mellitus, Sickle cell, Obesity, Physical inactivity, Hypercholesterolemia, Hypertension

## Abstract

Stroke is a devastating disease that occurs when a blood vessel in the brain is either blocked or ruptured, consequently leading to deficits in neurological function. Stroke consistently ranked as one of the top causes of mortality, and with the mean age of incidence decreasing, there is renewed interest to seek novel therapeutic treatments. The Scavenger Receptor Class B type 1 (SR-B1) is a multifunctional protein found on the surface of a variety of cells. Research has found that that SR-B1 primarily functions in an anti-inflammatory and anti-atherosclerotic capacity. In this review, we discuss the characteristics of SR-B1 and focus on its potential correlation with the modifiable risk factors of stroke. SR-B1 likely has an impact on stroke through its interaction with smoking, diabetes mellitus, diet, physical inactivity, obesity, hypercholesterolemia, atherosclerosis, coronary heart disease, hypertension, and sickle cell disease, all of which are critical risk factors in the pathogenesis of stroke.

## Background

Stroke is a devastating disease that affects populations around the world. Stroke is in the top three causes of mortality, and projections expect it to remain there in 2020 [1]. There are two primary categories of stroke: ischemic and hemorrhagic. Ischemic strokes result from clots that obstruct a blood vessel, preventing adequate supply to areas of the brain, and a hemorrhagic stroke occurs due to a ruptured blood vessel, leading to bleeding in the brain. Ischemic stroke comprises 80%–85% of all strokes, and the remaining 15%–20% are comprised of hemorrhagic strokes [2]. Maasz and Melegh state that the majority of ischemic strokes are the result of atherothrombosis (75%), whereas the remaining 25% of cases are from embolisms [2]. The dynamics of stroke epidemiology are rapidly changing. A study published by Feigin et al. regarding the global burden of ischemic and hemorrhagic stroke observed a threefold increase on the global burden from 1990 to 2013 [3]. Once previously considered a disease of the elderly, the incidence is increasing in young adults, thus renewing interest in its treatment and prevention [4–6]. A study involving stroke patients from China noted a striking decrease in the mean age of stroke onset in men from 1992 to 2014. In that timeframe, the mean age of stroke among men decreased by 0.28 years, and the incidence of first-ever stroke increased by 12% among men aged 45–64 [7]. However, Wang et al. did not observe a similar trend among women. Unsurprisingly, the risk factors for stroke in older populations also contribute to a significant proportion of stroke in younger adults [8]. The lifestyle choices of individuals across the globe subject them to an increased risk of stroke. While 9 out of 10 strokes stem from modifiable risk factors, prevention has still proven ineffective [9].

This article will review studies that have explored Scavenger Receptor Class B type 1 (SR-B1)’s role in well-known risk factors for stroke, such as smoking, diabetes mellitus, diet, physical inactivity, obesity, hypercholesterolemia, atherosclerosis, coronary heart disease, hypertension, and sickle cell disease.

## Scavenger Receptor Class B type 1

Research into scavenger receptors has spanned for several decades, with the first scavenger receptor being described in 1979 [10]. The scavenger receptors were divided and subdivided into “classes” and “types,” based on their sequences and variations in sequences as a result of alternative splicing, respectively [11]. These scavenger receptors are membrane-bound receptors, and while there are similarities in sequence among members of each class, different classes vary significantly in sequence similarity [12].

The class B scavenger receptors include SR-B1, SR-B2 (CD36), and LIMP2 [13]. These receptors are unique, in that they have two transmembrane domains flanking an extracellular loop, with cytoplasmic amino and C termini [13]. Other members of this class have shown promise in suppressing neuroinflammation, such as CD36, which also contributes to hematoma resolution [14, 15].

Scavenger Receptor Class B type 1 was identified as the first high-density lipoprotein (HDL) receptor, and it was shown to participate in the selective transport and regulation of cholesterol and lipids [16, 17]. This is significant, as HDL cholesterol levels are shown to be inversely correlated with a risk of stroke [18, 19]. However, ongoing research has uncovered SR-B1’s multifunctional capacity in recognizing and interacting with many ligands, including lipoproteins, apoptotic cells, cholesterol esters, phospholipids, proteoglycans, ferritin, and carbohydrates [12, 20, 21]. SR-B1’s other diverse functions also include pathogen recognition [22], protective effects against infertility in women [23], and lastly, efferocytosis of apoptotic cells, contributing to its anti-inflammatory characteristics [24]. These receptors are widely dispersed and expressed in various tissue and cell types, such as in the intestines, keratinocytes, epithelial cells, smooth muscle cells, monocytes, macrophages, mast cells, placenta, gallbladder, ocular tissues, endothelial cells, steroidogenic cells, astrocytes, neurons, hepatocytes, and adipocytes [16, 25–33]. The cell types that express SR-B1 and their functions pertaining to stroke and other neurological disorders have been summarized in Table 1.

**Table 1:** A summary of the cell types that express SR-B1, and their functions pertaining to the pathology of stroke and other neurological disorders

Cell type	Function	Reference
Epithelial cells	- Upregulates wound tissue repair by affecting the proliferative and migratory properties of keratinocytes - Regulates ceramide levels and maintain barrier function of keratinocytes - Vitamin E uptake might be regulated by SR-B1 in pneumocytes - Functions as plasma membrane cholesterol sensor	Muresan et al. [34], Muresan et al. [35], Kolleck et al. [36], Morel et al. [20]
Smooth muscle cells	- rHDL inhibits smooth muscle cell chemokine expression, p65, and proliferation through SR-B1 - HDL-associated lysosphinoglipids function to reduce ROS generation, which requires SR-B1 coordinate signaling	Van der Vorst et al. [37], Tolle et al. [38]
Monocytes	- Subclinical endotoxemia promotes atherosclerosis by converting monocytes into a persistent inflammatory state with reduced SR-B1	Geng et al. [39]
Macrophages	- SR-B1 invalidation reduces free-cholesterol-induced apoptosis and promotes atherosclerosis	Galle-Treger et al. [40]
Endothelial cells	- Functions as plasma membrane cholesterol sensor - Binding of SR-B1 with HDL activates endothelial NO synthase and stimulates endothelial cell migration - Apolipoprotein A-1 promotes endothelial repair through SR-B1 - SR-B1 acts as a mechanosensor in response to shear stress - SR-B1 is involved in transendothelial cholesterol transport	Saddar et al. [41], Yuhanna et al. 2001, Seetharam et al. [42], He et al. [43], Zhang et al. [44], Miao et al. [21]
Steroidogenic cells	- Estrogen increases brain SR-B1 levels - SR-B1 facilitates vasorelaxation pathway via interactions with DHEA-enriched HDL	Srivastava et al. [45], Paatela et al. [46]
Astrocytes	-Impairment of amyloid β uptake in Alzheimer’s correlated with a lower expression of SR-B1	Iram et al. [47]
Hepatocytes	- Mediates uptake of HDL-derived cholesterol ester	Acton et al. [17]

Recent research has uncovered anti-inflammatory characteristics of SR-B1 and functions through several pathways. Vascular smooth muscle cells expressing SR-B1 interact with HDL to inhibit p65 expression, a pro-inflammatory signal [37], and further interactions of HDL requires SR-B1 to partially inhibit reactive oxygen species generation [38]. Additionally, SR-B1’s role in inflammation is partly related to its function in facilitating clearance of other lipoproteins, such as low-density lipoproteins (LDL) and very low-density lipoproteins (VLDL) [48]. An increase in LDL plasma levels is recognized by the innate immune system and initiates an inflammatory response at the endothelial wall, where excess LDL is removed [49–51].

**Fig. 1:**
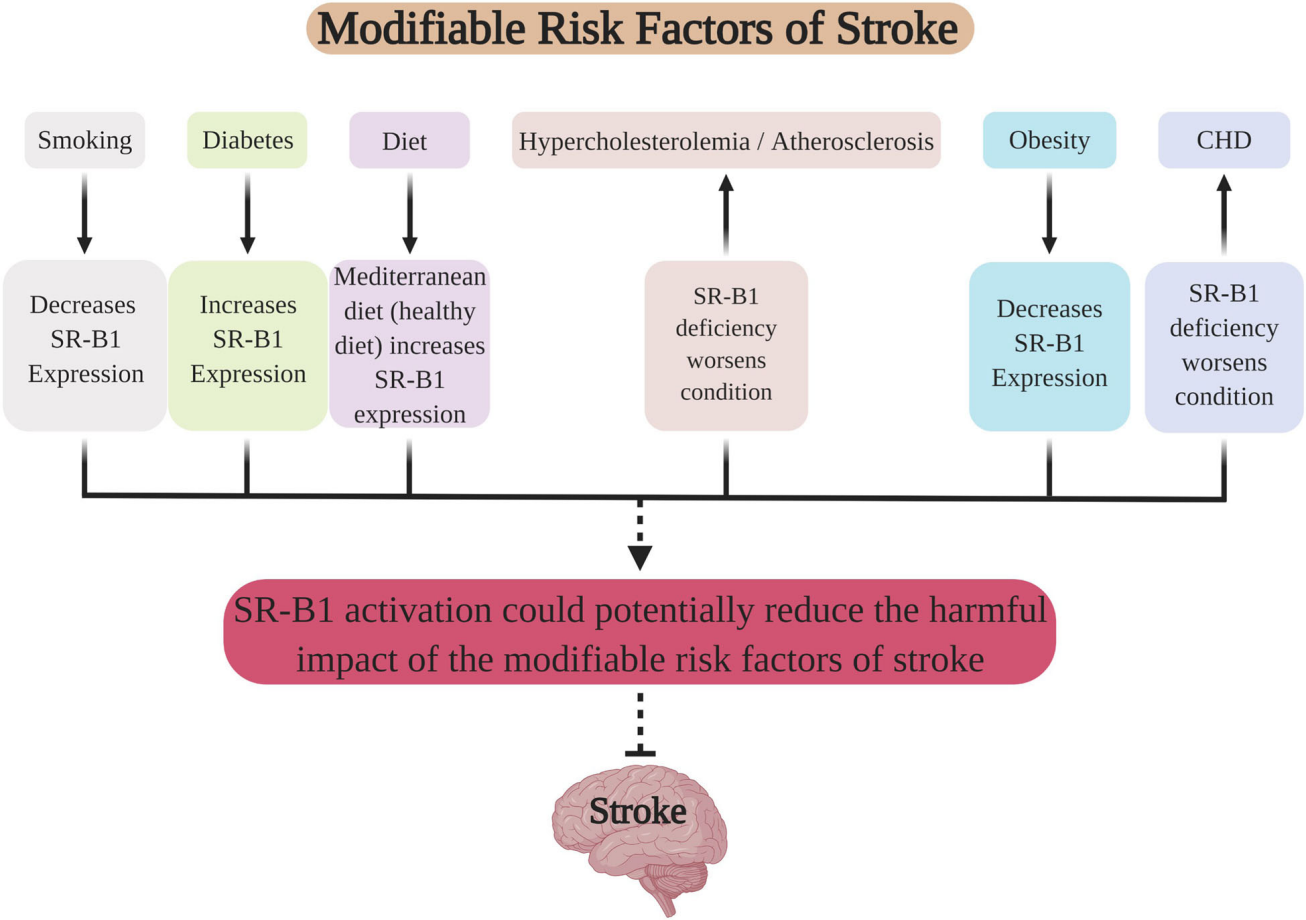
The relationship between primary modifiable risk factors and SR-B1. It suggests that the net effect of SR-B1 activation may provide a new strategy for stroke prevention/treatment

Emerging evidence has shown that the biological effect of SR-B1 is involved in multiple modifiable risk factors for stroke. Figure 1 depicts the relationship between primary modifiable risk factors of stroke and SR-B1. It suggests that the net effects of SR-B1 activation may provide a new strategy for stroke prevention/treatment. Table 2 summarizes specific details regarding the relationship that SR-B1 has with each identified modifiable risk factor.

**Table 2:** A detailed summary depicting the modifiable risk factors and their associated findings

SR-B1	Modifiable risk factors	Findings	References
Positive factors	Diet	- Mediterranean diet increases SR-B1 expression - SR-B1 has an important role in the uptake of lipid-soluble vitamins, which have been associated with lower risk of stroke - Chronic moderate alcohol accelerated cholesterol clearance via SR-B1-mediated reverse cholesterol transport	Nakamura et al. [52], Rimm et al. [53], Farras et al. [54], Han et al. [55] Li et al. [56]
	Exercise	- Increases SR-B1 expression	Wei et al. [57]
Negative factors	Smoking	- Smoking decreases SR-B1 expression of keratinocytes and possibly type II pneumocytes	Kolleck et al. [36], Sticozzi et al. [58]
	Diabetes	- Increases SR-B1 expression - SR-B1-knockout hyperglycemic mice had an increased incidence of coronary artery atherothrombosis, myocardial infarction, and early death	Hayashi et al. [59], Gonzalez et al. [60]
	Obesity	- Obesity-induced upregulation of miR-24 could function as a feedback regulator of SR-B1	Wang et al. [61]
	Hypercholesterolemia/atherosclerosis	- SR-B1 primarily functions in an atheroprotective capacity, when expressed in monocytes and macrophages	Kozarsky et al. [62], Zhang et al. [63], Van Eck et al. [64], Galle-Treger et al. [40]
	Coronary heart disease (CHD)	- SR-B1 deficiency leads to increased risk of CHD.	Zanoni et al. [65]
	Hypertension	- Lack of direct studies investigating SR-B1 expression in hypertension - Indirect effects may be possible, as a lack of SR-B1 led to impairment of nitric oxide, a potent vasodilator	Pearson et al. [66], Hermann et al. [67]
	Sickle cell	- No direct studies involving SR-B1 expression and sickle cell conditions - Indirect involvement is possible, as lipid dysregulation partially determines severity of sickle cell - SR-B1 is reported to facilitate cell-entry of malaria	Akinlade et al. [68], Rodrigues et al. [69]

## SR-B1 and smoking

A study by Epstein et al. reported a reduced likelihood of stroke, myocardial infarction (MI), or death over a period of 4.8 years, if patients quit smoking within 6 months after an ischemic stroke or transient ischemic attack (TIA) [70]. The effects of smoking have such an impact that a study by Hou et al. suggested that second-hand smoke increased the risk of death by 10% for all stroke types [71].

Cigarette smoke has an established record of damaging type II pneumocytes, epithelial cells that are found in lung tissue [72]. Notably, SR-B1 also has an important function in increasing vitamin E uptake from HDL into type II pneumocytes found within the lung [36]. This is significant, as a recent meta-analysis published by Cheng et al. found evidence that increased vitamin E intake was associated with a lower risk of stroke [73]. These type II pneumocytes function to secrete surfactant, which serves to reduce surface tension in the lung, which is necessary to prevent alveolar collapse after expiration [74]. This lung surfactant is comprised primarily of phospholipids, with the remainder of the composition consisting of proteins and neutral lipids [75]. Interestingly, another study determined that vitamin E is an integral component of lung surfactant assembly [76]. Furthermore, cigarette smoke can also alter the surfactant composition and function of this pulmonary surfactant [77]. One study also found that the reduced surfactant production induced by cigarette smoke was restored after the addition of vitamin E [78]. Dysfunction in a pulmonary surfactant caused by cigarette smoke unsurprisingly contributes to pathological dysfunction in other conditions of the lung as well, such as chronic obstructive pulmonary disease (COPD) [77, 79]. Additionally, a recent article suggests that COPD could contribute to the pathogenesis of stroke [80]. This avenue could be an area of interest in the future to determine if SR-B1’s role in facilitating vitamin E uptake in type II pneumocytes, which is then used to create pulmonary surfactant, plays a protective role in the presence of risk factors.

Additionally, cigarette smoke not only affects the lungs, but also the skin as well. It particularly affects keratinocytes, cells that function as a protective barrier against environmental damage [35]. Other skin conditions, such as psoriasis, which involves dysregulation of keratinocytes, have already been correlated with stroke [81]. Cholesterol comprises 25% of the outer layer of skin, known as the stratum corneum [58], and the SR-B1 receptor is also located on keratinocytes [34]. A study found that cigarette smoke dramatically decreased the expression of SR-B1 on keratinocytes. This decreased expression could contribute to an imbalance in cholesterol homeostasis. Their results suggested that the hydrogen peroxide found in cigarette smoke played a prominent role in the decreased expression of SR-B1 [58]. An in vitro study in 2018 also confirmed SR-B1’s role in modulating epidermal wound closure. The results suggested a new role for SR-B1, as a possible modulator of keratinocyte proliferation and migration via downregulation of nuclear cyclin D1 and active matrix metalloproteinase-9 expression [34]. Further evidence to support this claim was presented in a study by He et al., in which an apolipoprotein A-1 (Apo-A1) mimetic, known as 4F, acts on SR-B1 to promote endothelial repair, proliferation, and re-endothelialization in a carotid artery electric injury model [43].

After evaluating the evidence of the studies listed above, it is possible that a risk of stroke induced by smoking could be increased due to smoking’s harmful effects on SR-B1. Furthermore, SR-B1’s demonstrated function of injury repair on epithelial cells, such as keratinocytes, could also potentially contribute to the vascular repair of endothelial cells. More research is warranted to confirm this possible relationship.

## SR-B1 and diabetes mellitus

In the USA, diabetes is the seventh leading cause of death, and 65% of these deaths are attributable to cardiovascular disease, stroke, or both [82]. Globally, it is the third highest risk factor for premature mortality, and the cost of diabetes exceeds $174 billion USD [83, 84]. There are two types of diabetes mellitus, types 1 and 2. Type 1 diabetes mellitus is often considered a childhood illness, but the vast majority of the type 1 diabetics are adults [85]. As we learn more about type 1 diabetes, we begin to understand that it is more complex than we previously realized. It was once considered a single autoimmune disorder, but has now demonstrated that a variety of factors play an important role in type 1 diabetes, such as environmental factors, metabolism, immune systems, genome, and microbiome [86]. Type 2 diabetes is a global epidemic characterized by insulin resistance and is driven by modifiable risk factors, such as obesity, diet, and sedentary lifestyles, but is also subject to genetic predispositions and epigenetics [87]. In fact, stroke occurs twice as often in people with diabetes, compared to people without diabetes [88].

Unfortunately, both type I and type II diabetes lead to risk factors that contribute to stroke, as type I diabetics are more prone to hypertension, coronary heart disease, peripheral arterial disease, and stroke attributable to small-vessel disease [89]. Whereas, type II diabetics are more likely to have obesity, peripheral arterial disease, history of transient ischemic attack, and stroke attributable to large-artery atherosclerosis [89]. In a recent study, Gonzalez et al. [60] used streptozocin to induce hyperglycemia in SR-B1-knockout/hypoE mice. They reported that the SR-B1-knockout/hypoE hyperglycemic mice had an increased incidence of coronary artery atherothrombosis, myocardial infarction, and early death when on a high-fat, high-cholesterol diet [60]. SR-B1 was increased in type 2 diabetes patients, but not in those with hypercholesterolemia [90]. SR-B1 is upregulated in animal models of diet-induced insulin resistance [59].

Insulin induced a redistribution of SR-B1 from the cytoplasm to a predominantly perinuclear localization [59]. Hayashi et al. proposed the notion that this relocation of SR-B1 into the intracellular membranes is likely critical in SR-B1 function [59]. Their study found that response to the insulin-resistant state, thus leading to overproduction of intestinal apolipoprotein B48-containing particles. The significance of this is attributed to a recent study, in which the evidence suggested an increased fasting plasma apolipoprotein B48 as a risk factor for large artery atherosclerotic stroke [91].

These results suggest that SR-B1 could be upregulated in response to an insulin-resistant state, which may function as a protective mechanism for diabetes-induced complications.

## SR-B1 and diet

Diets play a fundamental role in the risk of stroke, heavily influencing it [92, 93]. Individuals who have western dietary patterns, which are associated with excessive saturated fats, processed grains, and simple sugars, are at an increased risk of stroke [94]. Conversely, other diets, such as the Mediterranean diet, have been shown to have the opposite effect. A Mediterranean diet consisting of staples such as fish, olive oil, fruits, vegetables, whole grains, legumes/nuts, and moderate alcohol consumption, as described by Widmer et al. [95], has been shown to significantly reduce the risk of stroke by more than 60% [96, 97].

Not surprisingly, the consumption of fish and alcohol, as well as regular exercise, are positively associated with HDL levels [52, 53, 98, 99]. Interestingly, olive oil and beans have also demonstrated effects in upregulating SR-B1 expression [54, 55, 100]. However, the effect of moderate alcohol use on stroke is still controversial. Alcohol has received mixed results pertaining to its risk associated with stroke. There have been documented effects of chronic moderate alcohol intake and its effect on SR-B1. Li et al. suggested that chronic moderate alcohol accelerated cholesterol clearance via SR-B1-mediated reverse cholesterol transport [56]. In this scenario, SR-B1 acts in a peroxisome proliferator-activated receptor gamma (PPARγ)-dependent manner, which coincides with other research regarding PPAR’s role in the regulation of lipid metabolism and insulin resistance [101].

An important function of the SR-B1 receptor involves the transport of lipid-soluble vitamins, K, A, D, and E. For several decades, vitamin K antagonists have been used extensively in the prevention of stroke, so it may seem counter-intuitive to consider promoting SR-B1, considering its role in the uptake of lipid-soluble vitamins. However, recent research suggests that SR-B1 may be a minor contributor to vitamin K absorption [102]. While a study by Jeyakumar et al. found that obese rats receiving normal levels of vitamin A in their diet showed high serum HDL and decreased hepatic SR-B1 expression levels when compared to their lean counterparts, SR-B1 was upregulated in the obese rats with a diet chronically supplemented with vitamin A [103]. Interestingly, 9-cis retinoic acid, a metabolite of vitamin A, has been shown to induce the expression of SR-B1 [104] and recently demonstrated a neuroprotective effect in a rat model of distal middle cerebral artery occlusion [105]. This study found that 9-cis retinoic acid significantly reduced body asymmetry, reduced neurological symptoms, and improved locomotor function in the rats after stroke. Other studies also support the notion that some lipid-soluble vitamins may offer benefits in stroke prevention or treatment. For example, vitamin D protects against atherosclerosis in settings of hypercholesterolemia [106]. As previously stated, SR-B1 is involved in vitamin E uptake in type II pneumocytes, but it also contributes to vitamin E transport across enterocytes [107], and a recent meta-analysis conducted by Cheng et al. has found that increased dietary vitamin E intake correlates with a reduced risk of stroke [73].

These studies suggest that many benefits of the Mediterranean diet could be mediated through SR-B1 activity. The multifunctional capacity of SR-B1 upregulation in lipid and cholesterol metabolism could potentiate its application for the treatment and prevention of stroke.

## SR-B1 and physical inactivity/obesity

Obesity is associated with a progressively increasing risk of ischemic stroke [108]. The epidemiology of obesity is unique, in that the epidemiology of obesity affects either middle-aged adults or all populations equally in low-income countries and high-income countries, respectively [109]. The scale of body weight is quantified as a body mass index (BMI). It is categorized as healthy weight, 18.5–24.9 kg/m^2^; overweight, 25.0–29.9 kg/m^2^; and obese, > 30 kg/m^2^. Unfortunately, some researchers expect the prevalence for being overweight and obese to reach dramatic levels in the upcoming years. For example, Keaver et al. [110] proposed that the overweight and obesity prevalence will reach 89% and 85% in males and females, respectively, by 2030 in Ireland [110]. Wang et al. showed that obesity-induced upregulation of miR-24 could function as a feedback regulator of SR-B1 in obesity [61]. Surprisingly, miR-24 induced different responses from SR-B1, depending on the cell type in which the receptor was located. It repressed HDL uptake in steroidogenic cells, but attenuated HDL uptake, lipid accumulation, and triglyceride levels in HepG2 cells [61]. Conversely, another study described a twofold to threefold increase in SR-B1 expression in exercising animals [57].

Obesity-induced repression of SR-B1 expression in some cells could potentially contribute to other risk factors. The findings that exercising upregulated SR-B1 expression, offers further support for the beneficial role of this receptor [57].

## SR-B1 and hypercholesterolemia/atherosclerosis

Hypercholesterolemia is a recognized causative agent in the development of atherosclerosis [111], a major risk factor for stroke [112]. Ongoing research has revealed a potential Janus-faced role of SR-B1 on atherosclerosis. Researchers have extensively studied the association between SR-B1 and cholesterol. Numerous studies suggest that SR-B1 has an inverse correlation with atherosclerosis [62–64, 113]. In fact, plasma-free cholesterol levels have increased sevenfold in SR-B1-knockout mice [114]. Other studies have suggested that mice deficient in SR-B1 have accelerated aortic sclerosis [40]. However, a recent article suggests that SR-B1 may have polarizing roles in atherosclerosis. Huang et al. presented a study implying that SR-B1 drives endothelial cell LDL transcytosis through a dynamic partnership with dedicator of cytokinesis 4, which will promote atherosclerosis [33]. Conversely, this same study provided conflicting information, in which they also found that the selective silencing of SR-B1 expression in hepatocytes resulted in more severe atherosclerosis and early death [33]. Likewise, Geng et al. suggest that in situations of chronic inflammation, monocytes can be programmed in a persistent state of inflammation, notably with reduced SR-B1 [39].

Propofol, a drug commonly used to induce and maintain anesthesia during surgery, has demonstrated anti-inflammatory characteristics, by decreasing free radical production [115]. A recent study demonstrated that propofol may promote expression of SR-B1 in macrophages via an enhancement of the PPARγ/liver X receptor alpha (LXRα) signaling pathway, suggesting that propofol could partially act via SR-B1 upregulation in the potential treatment of atherosclerosis [116].

Consequently, there are plenty of studies suggesting an atheroprotective role of SR-B1 activation, particularly in hepatocytes. Perhaps there is a positive systemic effect in its role in atherosclerosis. These studies appear to suggest that SR-B1’s role in atherosclerosis serves primarily in a protective capacity.

## SR-B1 and coronary heart disease/hypertension

Coronary heart disease (CHD) is the primary disease affecting the heart and is well-established as a critical burden on public health in developed countries [117–119]. Globally, hypertension occurs most frequently as a modifiable risk factor for stroke, in countries that are both developed and developing [120]. A study conducted in 2016 suggests an increased risk for CHD is associated with a genetic deficiency of SR-B1 [65]. Pearson et al. conducted a study with very intriguing results regarding SR-B1 and coronary dysfunction [66]. They explored the effects on the vasculature in mice that lacked the SR-B1, but also had hypomorphic apolipoprotein E while on a short-term high-fat Paigen diet. Furthermore, they found that the knockout of SR-B1 on endothelial cells with multiple diffuse coronary lesions displayed evidence that there was impairment of local vascular smooth muscle function. Ultimately, their results suggest that a lack of SR-B1 led to impairment of nitric oxide-mediated dilation of conductance and micro-vessels [66]. Hermann et al. elucidated on the fact that impaired nitric oxide bioactivity plays an important role in hypertension and cardiovascular disease [67]. Considering nitric oxide’s role as a potent vasodilator, there has not been any research published that directly explores the potential association between SR-B1 and hypertension. Further research is necessary to determine if a similar relationship exists between SR-B1 and hypertension.

## SR-B1 and sickle cell disease

Sickle cell disease is a widespread condition with a potentially severe prognosis. It has been well-established as a protective trait against malaria and is one of the most common severe monogenic disorders of the world [121, 122]. It is inherited in an autosomal recessive pattern, but has the severity is highly variable. This is in part due to the genetic variations that control hemoglobin F-gene expression, as well as the coinheritance of α-thalassemia gene [123]. Approximately 312,000 neonates are homozygous for hemoglobin S, the most common and clinically significant structural variant of hemoglobin and is responsible for the symptoms of sickle cell [124]. However, a study in 2014 confirmed that sickle cell patients undergoing a vaso-occlusive crisis had defective lipid metabolism [68]. Low levels of Apo-A1, a major structural apolipoprotein of HDL, also plays a role in the development of pulmonary arterial hypertension, a critical complication of sickle cell disease [125]. Regarding a possible connection between SR-B1 and sickle cell disease, it has been suggested that SR-B1 facilitates *Plasmodium* infection, and has been proposed as a potential target in malaria prophylaxis [69, 126]. However, there is a lack of published research to document whether a relationship, if any, is present between SR-B1 and sickle cell disease. There is insufficient evidence to conclusively suggest that SR-B1 is impacted by sickle cell.

## Conclusion

SR-B1 is a complex receptor affected by many comorbidities associated with stroke. The current literature shows that SR-B1 has both positive and negative effects. Some of the risk factors, such as smoking and obesity, act to inhibit the activity of SR-B1, whereas other studies of modifiable risk factors, such as diabetes, hypercholesterolemia, atherosclerosis, and CHD, suggest that a lack of this receptor exacerbates the condition. Interestingly, Mediterranean diets, exercise, and diabetes promote its activity. It is possible that an upregulation in diabetes could be acting as an endogenous protective response, but more research is necessary to validate this speculation. Although we presented evidence for possible indirect involvement with hypertension and sickle cell, it is to be noted that there is a lack of research defining a specific association between SR-B1 and other risk factors for stroke, such as hypertension, atrial fibrillation, peripheral artery disease, and sickle cell.

Considering the extensive involvement of SR-B1 in the various modifiable risk factors of stroke, there is likelihood that the net effect of SR-B1 activation may favor the prevention/treatment of stroke (Fig. 1). Further research is warranted to obtain more information regarding SR-B1’s role in stroke and determine whether it serves as a common denominator between the various modifiable risk factors.

## Data Availability

Not applicable

## Abbreviations

APO-A1: Apolipoprotein A-1
BMI: Body mass index
CHD: Coronary heart disease
COPD: Chronic obstructive pulmonary disease
HDL: High-density lipoprotein
IFNα: Interferon α
IL-10: Interleukin-10 TGF**-**β Transforming growth factor β
LDL: Low-density lipoprotein
LXRα: Liver X receptor alpha
MI: Myocardial infarction
PPARγ: Peroxisome proliferator-activated receptor
ROS: Reactive oxygen species
SR-B1: Scavenger Receptor Class B type 1
TIA: Transient ischemic attack
VLDL: Very low-density lipoprotein
